# A Translational Perspective of Maternal Immune Activation by SARS-CoV-2 on the Potential Prenatal Origin of Neurodevelopmental Disorders: The Role of the Cholinergic Anti-inflammatory Pathway

**DOI:** 10.3389/fpsyg.2021.614451

**Published:** 2021-03-31

**Authors:** José Javier Reyes-Lagos, Eric Alonso Abarca-Castro, Juan Carlos Echeverría, Hugo Mendieta-Zerón, Alejandra Vargas-Caraveo, Gustavo Pacheco-López

**Affiliations:** ^1^Faculty of Medicine, Autonomous University of the State of Mexico (UAEMex), Toluca, Mexico; ^2^Multidisciplinary Research Center in Education (CIME), Autonomous University of the State of Mexico (UAEMex), Toluca, Mexico; ^3^Basic Sciences and Engineering Division, Campus Iztapalapa, Metropolitan Autonomous University (UAM), Mexico City, Mexico; ^4^Health Institute of the State of Mexico (ISEM), “Mónica Pretelini Sáenz” Maternal-Perinatal Hospital, Toluca, Mexico; ^5^Biological and Health Sciences Division, Campus Lerma, Metropolitan Autonomous University (UAM), Lerma, Mexico

**Keywords:** cholinergic anti-inflammatory pathway, quality of life, COVID-19, neurodevelopmental disorders, human development, heart rate variability, SARS-CoV-2, maternal immune activation

## Abstract

The emergent Coronavirus Disease 2019 (COVID-19) caused by the Severe Acute Respiratory Syndrome Coronavirus 2 (SARS-CoV-2) could produce a maternal immune activation (MIA) via the inflammatory response during gestation that may impair fetal neurodevelopment and lead to postnatal and adulthood mental illness and behavioral dysfunctions. However, so far, limited evidence exists regarding long-term physiological, immunological, and neurodevelopmental modifications produced by the SARS-CoV-2 in the human maternal-fetal binomial and, particularly, in the offspring. Relevant findings derived from epidemiological and preclinical models show that a MIA is indeed linked to an increased risk of neurodevelopmental disorders in the offspring. We hypothesize that a gestational infection triggered by SARS-CoV-2 increases the risks leading to neurodevelopmental disorders of the newborn, which can affect childhood and the long-term quality of life. In particular, disruption of either the maternal or the fetal cholinergic anti-inflammatory pathway (CAP) could cause or exacerbate the severity of COVID-19 in the maternal-fetal binomial. From a translational perspective, in this paper, we discuss the possible manifestation of a MIA by SARS-CoV-2 and the subsequent neurodevelopmental disorders considering the role of the fetal-maternal cytokine cross-talk and the CAP. Specifically, we highlight the urgent need of preclinical studies as well as multicenter and international databanks of maternal-fetal psychophysiological data obtained pre-, during, and post-infection by SARS-CoV-2 from pregnant women and their offspring.

## Introduction

During pregnancy, there is an increased risk of infection by viruses and bacteria (Silasi et al., 2015; Barinov et al., 2020). Throughout this unique physiological condition, pregnant women are susceptible to gestational infections owing to immunomodulatory changes that naturally occur in their bodies, allowing the implantation and growth of the fetal allograft (Hedge, 1991). Pregnancy was early considered as a high-risk condition for contracting the COVID-19 disease (Phoswa and Khaliq, 2020); approximately 200 million pregnant women were estimated as having a potential risk of SARS-CoV-2 infection worldwide (Vogel, 2020). It could be presumed that the gestational period makes difficult the clinical course of COVID-19 and thus become related with an increased mortality rate.

Cardinal clinical manifestations of patients with COVID-19 disease include fever, cough, and dyspnea (Harapan et al., 2020; Knight et al., 2020; Rodriguez-Morales et al., 2020). Among these patients, the most significant comorbidities are hypertension, cardiovascular disease, and diabetes (Rodriguez-Morales et al., 2020). In pregnant women, recent evidence has shown that the occurrence of COVID-19 during gestation is mainly associated with higher preterm birth rates (Allotey et al., 2020). Yet, an increased number of intrauterine fetal and neonatal deaths, premature rupture of membranes, and miscarriages as well as a reduction of fetal movements have also been documented in a systematic review (Amaral et al., 2020).

**Figure 1:**
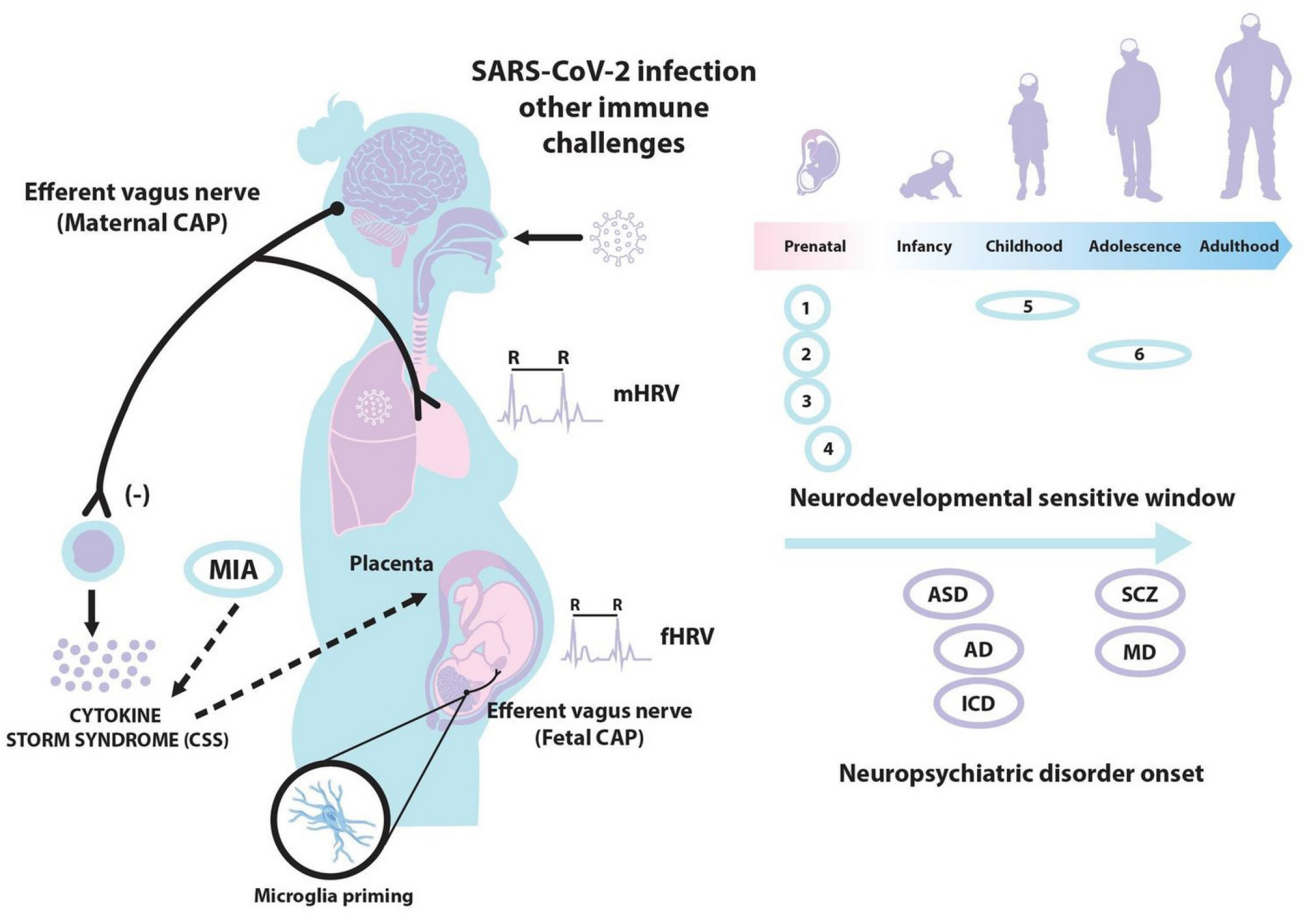
A gestational infection triggered by SARS-CoV-2 may produce a maternal immune activation (MIA) and cytokine storm syndrome (CSS). The activation of the maternal and/or fetal cholinergic anti-inflammatory pathway (CAP) via the efferent vagus nerve and transplacental interactions could downregulate such CSS. The analysis of maternal and fetal heart rate variability (mHRV and fHRV) might be considered for assessing maternal and fetal CAP activation. Also, a gestational infection by SARS-CoV-2 may produce fetal neuroinflammation and microglial activation, increasing the risks of several neurodevelopmental disorders in the offspring and even an altered long-term cognitive function in adulthood: (1) neuronal migration; (2) axonal and dendritic growth; (3) programmed cell death; (4) synaptogenesis; (5) myelination; and (6) process modeling/synaptic refinement. ASD, autism spectrum disorder; SCZ, schizophrenia; AD, anxiety disorders; MD, mood disorders; ICD, impulse-control disorders.

The COVID-19 pandemic has forced to establish priority in the short-term mitigating health systems mechanisms. However, owing to the pandemic recent upsurge, no evidence has been reported concerning the middle- and long-term neuroimmune perinatal alterations. Specifically, the effects caused by this disease in vulnerable groups, such as pregnant women and their offspring. Some authors have already proposed that this disease could alter fetal neurodevelopment and even postnatal life (Liu H. et al., 2020). With this perspective article, we first describe the principle of a potential maternal immune activation (MIA) by SARS-CoV-2, as well as the maternal response and inflammation occurred in response to this severe infection. Next, we consider the possibility of neurodevelopmental disorders of the offspring in response to a COVID-19 disease. Additionally, we introduce a possible relationship between the cholinergic anti-inflammatory pathway (CAP), a complex neuroimmune mechanism (Huston and Tracey, 2011), and COVID-19 in pregnancy. Finally, we discuss some autonomic measures, such as those derived from the analysis of maternal and fetal heart rate variability (HRV), which may contribute to our current understanding of COVID-19 in a perinatal translational research. The hypothesized associations among SARS-CoV-2, MIA, the CAP, and neurodevelopmental disorders are shown in Figure 1.

Some specific examples and evidence concerning MIA, CAP, and neurodevelopmental disorders are presented in the following subsections, whereas our final hypothesis and opinions linking these topics are mainly addressed in the perspective section.

## MIA and SARS-CoV-2

Infections during pregnancy can cause disturbances in fetal neurodevelopment by the elevation of pro-inflammatory cytokines in the maternal host. Interleukin 6 (IL-6) has been described as a key molecular mediator for the early pathophysiological mechanisms that predispose to neuropsychiatric disorders such as schizophrenia and autism (Smith et al., 2007). Actually, a link between influenza outbreaks and increased risks for schizophrenia in the offspring has been already reported (Tochigi et al., 2004). In a mouse model of influenza infection in pregnancy, an upregulation of the serotonin 5-HT_2A_ receptor in the offspring cortex is observed, which is similarly reported in the postmortem prefrontal cortex from schizophrenic patients (Saunders et al., 2020). In other preclinical models of MIA, the activation of the innate immunity of a pregnant female mouse is mediated by the Toll-like receptor 3 (TLR-3) pathway through the use of a synthetic immunogen, poly-inosinic acid: poly-cytidylic (Poly I:C), which is a mimetic molecule of the replicating genome of single-stranded RNA viruses. This molecular pattern of a double strand of RNA is recognized by the TLR-3 of host innate immune cells, inducing the activation of the transcriptional factor NF-κB that promotes the expression of inflammatory cytokines and the production of type 1 interferons (Alexopoulou et al., 2001). Similarly, several infections of other single-stranded RNA genome viruses such as SARS-CoV and the Middle East Respiratory Syndrome Coronavirus (MERS-CoV) have also been found to activate TLR pathways, specifically that of the TLR-3, generating aberrant signaling through inflammatory cytokines, chemokines, and interferons (Totura et al., 2015).

It has been consistently documented that the infection by SARS-CoV-2 generates a complex production of molecules of the inflammatory response. For example, increased levels of cytokines (IL-6, IL-10, and TNF-α), lymphopenia in T cells (CD4 + and CD8 +), and a decreased expression of IFN-γ in CD4 + T cells have all been reported (Pedersen and Ho, 2020). These immunological changes are even associated with the severity of COVID-19 disease (Pedersen and Ho, 2020). Similarly, some patients with SARS-CoV-2 have shown a TH17 cytokine profile, contributing to the cytokine storm syndrome (CSS) (Ur and Verma, 2020; Ye et al., 2020). Notwithstanding that several cytokines are involved in such TH17 cell response, the most relevant is the surge of IL-17 that is responsible for granulopoiesis and neutrophil recruitment (Wu and Yang, 2020). This evidence suggests that patients with severe COVID-19 manifest an uncontrolled excessive inflammatory response, characterized by a hyperinflammatory response to SARS-CoV-2 that is facilitated by a possible unregulated immune system of the host. At present, few clinical studies have been carried out to explore the manifestation and the effects of CSS by COVID-19 during human pregnancy. Nonetheless, some case studies showing respiratory diseases, abnormal Apgar indexes, and pneumonia have already been reported in neonates born from SARS-CoV-2-infected women (Nayak et al., 2020; Wu et al., 2020).

Furthermore, relevant evidence documented that the transplacental transmission of SARS-CoV-2 infection is possible during the last weeks of pregnancy (Vivanti et al., 2020). Recent pathology findings in placentas of SARS-CoV-2-infected cases show mononuclear cell inflammation of the intervillous space, which is accompanied by a syncytiotrophoblast necrosis (Schwartz and Morotti, 2020). In addition to placental inflammation and neonatal viremia, the transfer of pro-inflammatory cytokines across the placenta could lead to fetal brain cortical malformations, and changes in macrophages function that may be even sustained up to adulthood in accordance with the so-called “*Barker hypothesis of the fetal origin of adult disease*” (Barker, 2001). Novel preclinical findings indicate that MIA alters fetal brain development, with implications for long-term cognitive function and behavioral phenotype (Baines et al., 2020; Easterlin et al., 2020).

## Neurodevelopmental Disorders and COVID-19

Findings derived from epidemiological and preclinical models also show that MIA caused by a viral infection might be linked to an increased risk of neurodevelopmental disorders in the offspring, such as the autism spectrum disorder, schizophrenia, and depression (Pacheco-López et al., 2013; Meyer, 2014; Al-Haddad et al., 2019). Recent studies have even postulated that the dietary intake of anti-inflammatory nutrition in pregnant women with COVID-19 infection and their children could help reduce the risk of neuropsychiatric disorders (Hashimoto, 2020). Other evidence indicates that a severe crisis or traumatic situation in childhood may lead to difficulties in psychological regulation (Thabrew et al., 2012) and increase the risk of diseases during adult life (Vargas, 2012). In the antenatal and early childhood stages, these episodes can cause adverse effects to the subjects, who are supposed to live in a stable and protective environment (López Soler, 2008). Therefore, an irruption of the life cycle at any of such stages may generate long-term repercussions in different areas of human development, its capacities, and corresponding quality of life (United Nations Development Programme, 2014).

Some studies show that critical or stressful situations during pregnancy generate posterior difficulties in childhood. For example, external environmental factors such as domestic violence, other crises, and psychological trauma affect pregnancy outcomes (López Soler, 2008; Vargas, 2012). COVID-19 should thus act as a complex insult for pregnant women. In fact, knowing a positive diagnostic test result of COVID-19 may generate important physical and emotional implications in pregnant women who may become anxious in identifying themselves as sick people at risk (Bermejo-Sánchez et al., 2020; Lorenzo Ruiz et al., 2020). Different studies have shown that the presence of a variety of psychiatric disorders in adulthood has been associated with early traumatic experiences (Ordóñez-Camblor et al., 2016). Events in early life and childhood alter the neurodevelopment, the dynamic gene-environment interplay, and the programming of the body's neurological, immune, and endocrine systems (National Research Council (US) Institute of Medicine (US) Committee on Integrating the Science of Early Childhood Development, 2000). We consider that this neuro-immune-endocrine insult, as COVID-19, has long-lasting implications for subsequent trajectories of human development (United Nations Development Programme, 2014).

Pregnant women with confirmed COVID-19 infection should be closely supervised and monitored to be able to early recognize any clinical deterioration of the mother and fetus. In this context, we propose, as one possible approach to facilitate this follow-up, to monitor both maternal and fetal neuro-immune interactions by assessing the CAP (Gallowitsch-Puerta and Pavlov, 2007).

## The Cap and COVID-19

Our knowledge about the neuroimmune changes that exists in the maternal-fetal binomial owing to a COVID-19 infection is minimal; however, some homeostatic mechanisms such the maternal or fetal CAP could be crucial or become altered during pregnancy. The CAP is a complex neuroimmune homeostatic mechanism that suppresses pro-inflammatory cytokine release via the vagus nerve (Huston and Tracey, 2011; Ur and Verma, 2020). The nucleus tractus solitarii integrates the CAP with other immunomodulatory responses because it spreads afferent vagal nerve neuroimmunomodulation signals to the brain (Pavlov et al., 2003). Specifically, an inflammation of the nucleus tractus solitarii can cause a disruption of the CAP and the hypothalamic–pituitary–adrenal axis resulting in a CSS (Ur and Verma, 2020).

In pregnant women, we hypothesize that the activation of the maternal and fetal CAP and the placental interactions might modulate the neuroimmune mechanisms employed to mitigate the CSS, possibly reducing tissue and cell damages, and potentially minimizing the effects of a SARS-CoV-2 infection (Kreis et al., 2020). Interestingly, recent studies have proposed that some severe COVID-19 manifestations could be linked to an impairment of the CAP (Farsalinos et al., 2020). Some authors even consider that the administration of a cholinergic agonist (e.g., pyridostigmine) could inhibit the inflammatory response and lower the mortality of COVID-19 patients (Ahmed, 2020). A disruption of the CAP may thus cause or exacerbate the severity of the maternal and fetal CSS. Interestingly, some preclinical evidence shows that a decreased fetal neuroinflammation is correlated with higher vagus nerve activity fluctuations in near-term ovine fetuses (Frasch et al., 2016).

The CAP may be more relevant in a SARS-2-CoV infection compared with other physiological mechanisms because further evidence indicates that the vagus nerve plays a relevant role in pulmonary inflammation (dos Santos et al., 2011). A detailed description of the neuroimmune mechanisms related to a possible CAP and its impairment in the case of this infection can be found elsewhere (Leitzke et al., 2020; Liu W. et al., 2020). In SARS-CoV-2 infections, young patients generally experience mild symptoms, while fatal interstitial pneumonia is more frequently reported in older patients. It is known that vagal activity declines with normal aging (De Meersman and Stein, 2007), and some authors have already suggested that a deregulated CAP can be associated with the severe manifestations of COVID-19 disease (Farsalinos et al., 2020). Thus, we speculate that an attenuated maternal CAP could also lead to a worse prognosis of COVID-19 in pregnant women and their offspring.

In pregnancy, preclinical studies support a presumed link between cholinergic signaling in fetal brain microglia and the inflammatory state, suggesting the possibility that early disruptions in microglial iron metabolism may impair fetal neurodevelopment (Cortes et al., 2017). Owing to the changing nature of human development from childhood to adulthood, and the participation of peripheral macrophages in the synaptogenesis of brain's microglia as well as innate immune responses, other authors consider that the effects of a permanent peripheral cholinergic activation can have an effect on the programming of the brain wiring and immune function (Frasch et al., 2018).

Additionally, other findings suggest that fetal CAP activity via vagal nerve stimulation using agonists of the α7 subunit of the nicotinic acetylcholine receptor (α7nAChR) abrogates the activation of brain astrocytes and microglia, thereby restoring their physiological immunometabolic phenotype and thus preventing a sustained switch to a reactive phenotype with decreasing glial priming (Frasch et al., 2019). We speculate that a disrupted fetal or maternal CAP during COVID-19 disease may thus contribute to fetal neuroinflammation, prenatal stress, and even preterm labor.

A non-invasive psychophysiological approach to indirectly evaluate the activity of the autonomic nervous system is the analysis of HRV, which has been considered as an important “window” for understanding the neuroimmune interactions involving the vagus nerve (Huston and Tracey, 2011) and also the response to inflammatory processes (Williams et al., 2019). Thus, we propose the analysis of maternal and fetal HRV as a non-invasive, economic, and quantitative approach to reliably assess the maternal and fetal CAP and the potential MIA produced by COVID-19 disease during human pregnancy.

## Perspective

Given the possible effects on neurodevelopment that can affect human development and the quality of life, the understanding of the maternal and fetal vagal–immune pathway during a gestational infection by SARS-CoV-2, and exploring the short- and long-term psychophysiological condition of the offspring, warrant special attention. This knowledge demands the use of non-invasive and economic physiological measures to assess fetal and maternal cardiac vagal activity, such as the maternal and fetal HRV. In this context, the use of a vagal nerve stimulation (VNS) for treating COVID-19 has also been proposed (Bonaz et al., 2020; Fudim et al., 2020). By activating the vagus nerve, it has been demonstrated positive therapeutic effects on COVID-19 symptoms via anti-inflammatory mechanisms (Mazloom, 2020). Further preliminary observations suggest that VNS provides clinical benefits in patients with this disease (Staats et al., 2020). Our main perspective is that the continuous monitoring of maternal and fetal vagal activity by HRV would also be an innovative application for managing COVID-19 patients.

Currently, there is insufficient evidence of the relationship among MIA, SARS-CoV-2, the CAP, and neurodevelopmental disorders. Our manuscript aims to offer a brief perspective and hypothesizes about a potential MIA by SARS-CoV-2, which could lead to neurodevelopmental disorders. We have also considered a cytokine cross-talk between the mother and the fetus as well as the role of the CAP (Figure 1). We highlight the urgent necessity of creating multicenter and international databanks of maternal-fetal psychophysiological signals, obtained pre-, during, and post-infection by SARS-CoV-2 from pregnant women. Preclinical studies over long enough periods should also be conducted, allowing these disorders to appear. Other authors support the consideration that health indicators of maternal, neonatal, and subsequent development should be also monitored after *in-utero* exposure to SARS-CoV-2 (Easterlin et al., 2020). Furthermore, longitudinal recordings and offspring neurodevelopmental assessments including immunological markers are desirable to appraise long-term effects. We anticipate that the collection of such multivariate data will help to understand the neuroimmune mechanisms activated by the SARS-CoV-2 infection from a translational perspective in both pregnant women and offspring.

## Author Contributions

JR-L: conceived and designed the document and writing—review and editing. EA-C, JE, HM-Z, and AV-C: writing—review and editing. GP-L: conceived and designed the document, writing—review and editing, and supervision. All authors contributed to the article and approved the submitted version.

## Conflict of Interest

The authors declare that the research was conducted in the absence of any commercial or financial relationships that could be construed as a potential conflict of interest.
